# Osteoradionecrosis of the jaw: A mini review

**DOI:** 10.3389/froh.2022.980786

**Published:** 2022-07-28

**Authors:** Annu Singh, Joseph M. Huryn, Kenneth L. Kronstadt, SaeHee K. Yom, Joseph R. Randazzo, Cherry L. Estilo

**Affiliations:** Dental Service, Department of Surgery, Memorial Sloan Kettering Cancer Center, New York, NY, United States

**Keywords:** osteoradionecrosis (ORN), head and neck cancer, osteoradionecrosis of jaw, intensity modulated radiation therapy (IMRT), oral complications of cancer therapy

## Abstract

Osteoradionecrosis (ORN) of the jaw is one of the most dreaded complications of head and neck radiation therapy. Despite the evolution of radiation treatment modalities, ORN continues to remain a therapeutic challenge and its etiopathogenesis still remains unclear. It is clinically characterized by exposed necrotic bone within the head and neck radiation field. Over the past years, several studies have reported on the definition, staging, incidence, etiology, and management of this oral complication. In this review, we summarize the literature on ORN and discuss our institutional experience and management strategies that aim to predict and mitigate risk for ORN.

## Introduction

Radiation therapy (RT) plays a key role in the management of head and neck cancers resulting in improved tumor control and increased survival rates [1]. Despite these advances, patients treated with RT often develop radiation-associated toxicities such as osteoradionecrosis (ORN) [2, 3]. In this condition, bone within the radiation field becomes devitalized and exposed through the overlying skin or mucosa that persist as a non-healing area. The history of ORN dates back 100 years when it was first noted by Regaud in 1922 [4]. Although rare, ORN is one of the most dreaded complications of head and neck RT that can significantly impact quality of life [5, 6]. Review of the literature over the past 100 years showed improvement in the prevalence of ORN. This could be attributed to the technological advancements of radiation modalities, clinicians' awareness, patient education, improvement in recognizing and mitigating risk factors and cautious approach in the dental management post head and neck RT. In this review, we summarize the literature on ORN and discuss our institutional experience and management strategies that aim to predict and mitigate risk for ORN.

## Methods

An electronic search of PubMed was performed using the keyword “osteoradionecrosis” to identify literature published in English between January 1922 and April 2022 which revealed 2740 publications. Following the search results, relevant publications that focused mainly on “osteoradionecrosis of the jaw” were carefully reviewed, the most significant information was collected and compiled in this mini review.

## Review of the condition

### Diagnosis

The bone changes associated with head and neck RT was first described as “radiation osteitis” [7]. Since then, numerous terms and definitions have been used to describe ORN with subtle differences based on the clinical presentation and duration of condition [8, 9].

Based on the consensus, clinical diagnostic criteria of ORN are as follows [10–12]:

- The affected site is within the head and neck radiation field.
- Mucosal breakdown or failure to heal occurs, resulting in bone exposure.
- The overlying bone is “dead” or necrotic.
- The bone exposure persists for a minimum 3 months.
- There is an absence of recurrent tumor/metastases on the affected site.

Although these criteria are widely accepted for a clinical diagnosis, they fail to incorporate the radiographic evidence of ORN with intact mucosa [13, 14].

### Staging

Likewise, various staging systems for ORN have been published for routine clinical practice and management. Marx's staging system is based on response to hyperbaric oxygen (HBO) therapy and the need for subsequent surgical intervention [10]. The other classifications were based on various criteria, including clinical–radiological findings, disease progression, degree of bone damage, duration of bone exposure, oro-cutaneous fistulae, pathological fracture, and management [9, 15]. A recent study has quantified ORN in terms of hard and soft tissue involvement [16]. Notani's classification is a simple system based on anatomical boundaries [17]:

Stage I ORN is confined to alveolar bone.

Stage II ORN is limited to the alveolar bone and/or above the level of the inferior alveolar canal. Stage III ORN is under the lower part of the inferior alveolar canal, with fistula or bone fracture.

A recent study has modified the Notani's ORN classification incorporating minor bony spicule measuring <20 mm^2^ that is seen as a common outcome in clinical trials [18]. The authors believe that this modification might be most suitable for prospective interventional trials of ORN prevention or treatment. NCI Common Terminology Criteria for Adverse Events (CTCAE) also includes “osteonecrosis of mandible” as a musculoskeletal and connective tissue disorder and consider mainly its functional impact [19]. This staging system has been used in recent studies reporting ORN toxicity following proton radiation therapy [PRT] [20, 21]. A recent study has applied the American Academy of Maxillofacial Surgeons classification system, commonly used for medication-related osteonecrosis of the jaw, in analyzing the severity of ORN in head neck cancer patients [22].

### Signs and symptoms

**Figure 1:**
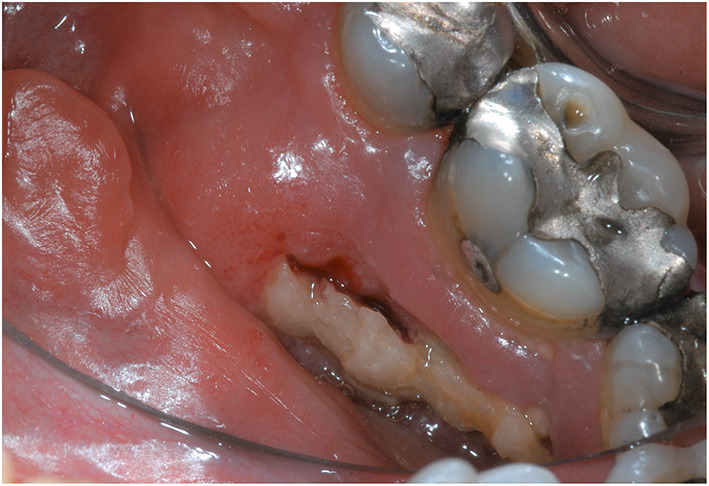
A 57-year-old male patient, diagnosed with HPV positive T2N2c squamous cell carcinoma of left base of the tongue and treated with definitive chemoradiation (6996cGy in 33 fractions), developed a spontaneous exposed bone measuring 1.5 × 0.5 cm in the lingual posterior mandible adjacent to right mandibular first and second molars, consistent with Notani Stage II ORN. The exposed bony edges were sharp causing irritation to adjacent soft tissues.

Although ORN manifestation varies greatly, clinical sign typically includes an area of exposed bone area (Figure 1) or a fistula that probes to bone. Tooth mobility or spontaneous tooth exfoliation can also be an indication for ORN. Several cases of “radiographic” ORN with unexposed bone necrosis and intact mucosa have also been reported [14]. Radiographic signs can range from localized osteolytic areas, extensive osteolytic areas, sequestrum and mandibular fracture as seen on a panoramic radiograph [23]. ORN can present as radiolucent areas surrounding the extraction sockets that remain visible for more than 12 months [9]. Computed tomography scan can depict ORN lesions as osteolytic lesions or cortical erosions involving the buccal or lingual surface and often associated with bone fragmentation [23]. Early stages of ORN can be asymptomatic [8]. However, pain, with or without swelling, is a common symptom associated with ORN. Poor oral hygiene and food impaction within the exposed bone area may also be present [8, 12, 24]. Patients may present with sensory neurological symptoms such as dysesthesia, or anesthesia in the distribution of the inferior alveolar nerve in the mandible in late stage ORN. As ORN progresses, patients may develop trismus, neuropathic pain, and other symptoms such as secondary infection resulting in chronic pus drainage, draining extra oral fistulae or even pathological jaw fracture [25].

### Pathophysiology

ORN has a known predilection for the mandible over maxilla [26]. This vulnerability may be due to its relative hypovascular nature and proximity to the primary tumor causing inevitable radiation exposure within the radiation field. The posterior mandible is more commonly affected because of its high bone density resulting in an increased absorption of radiation dose. [27]. Multiple theories have been postulated regarding the etiopathogenesis of ORN, but the exact mechanism is complex and poorly understood [8]. Early studies showed evidence of bacteria in tissues affected by ORN as well as microscopic tissue changes [28]. This was popularized by Meyer who proposed the classic triad sequence of pathogenesis as “radiation, trauma, and infection” [29]. He believed that ORN resulted from secondary infection due to local injury to the devitalized bone resulting in “radiation induced osteomyelitis.” This theory explained the role of antibiotic therapy in ORN management. Based on the evidence of ORN in the absence of trauma (extraction), Marx proposed a new theory that was attributed to the radiation-induced endarteritis resulting “hypoxia, hypovascularity, and hypocellularity” [10]. Driven by his theory that persistent hypoxia can cause a chronic non-healing wound, his hypothesis formed the cornerstone for the use of hyperbaric oxygen (HBO) in the treatment of ORN. A current theory proposes that ORN occurs by a “radiation-induced fibro atrophic mechanism” whereby the activation and dysregulation of fibroblastic activity leads to atrophic tissue within a previously irradiated area [30]. To reverse these changes, new therapeutic regimens have been developed wherein pentoxifylline and tocopherol (vitamin E) act synergistically as potent antifibrotic agents [31].

### Prevalence

The prevalence of ORN varies widely in the literature ranging from 0.4 to 56% [9]. There is an approximately 20% decrease in the rate of ORN from earlier decades [32, 33] to 4–8% in modern era [34, 35]. This overall reduction of ORN can be attributed to the evolution in radiation modalities from the conventional/2D RT to 3-D conformal RT to intensity modulated radiation therapy (IMRT) [36, 37]. One study also reported lack of mandibular ORN in head neck cancer patients following IMRT with the use of a strict prophylactic dental care policy [38]. A retrospective study from our own institution reported an incidence of 4.3% over a ten-year period in 1023 patients oral and oropharyngeal cancers with IMRT [39]. Proton radiation therapy (PRT) allows further conformal treatment volumes and greater tissue-sparing capability in head and neck radiation due to its inert property of Bragg Peak [40]. This technique includes a smaller volume of the jaw that receives high irradiation doses thus potentially decreasing the likelihood of ORN [41]. Zhang et al. reported reduced incidence of ORN in oropharyngeal cancer patients: 2 vs 7.7% when treated with PRT as compared to IMRT [20].

### Time lapse to ORN

ORN can occur at any time, even beyond 10 years following RT [42, 43]. A retrospective study reported that the median time interval between RT and development of ORN was 13 months (range, 2–122 months) [26]. However, it is most frequently noted (70–94%) in the first few years after completion of RT [42, 44]. The median latency period is usually reported as 12–24 months [39, 45]. Early onset ORN occurring within 24 months after RT is thought to be related to radiation doses higher than 60Gy; it can develop spontaneously or following dentoalveolar trauma. In contrast, late onset ORN is thought to arise from trauma in a chronically hypoxic tissue environment [42, 46]. A systematic review described increased risk of ORN following post-radiation extraction in the time period of 2–5 years after RT [47]. In a retrospective study of treated with IMRT in our institution, ORN developed earlier in patients with oropharyngeal cancer (median, 14.6 months) than those with oral cavity cancer (median, 36.1 months) [39].

### Management

The management of patients with ORN varies considerably and depends on the severity of the complication [48]. Conservative approaches are generally reserved for asymptomatic or mildly symptomatic patients (Notani I or II) [49, 50]. This includes close observation, strict oral hygiene maintenance, saline irrigation and chlorhexidine mouth rinses, systemic antibiotic therapy for acute infections, anti-inflammatory and analgesics when necessary, avoidance of local irritants like tobacco and alcohol use, discontinuation of ill-fitting dentures. Simple surgical intervention involves smoothening of sharp bony edges to prevent traumatic ulcerations to adjacent soft tissues and gentle debridement of mobile bony sequestrum. Fixation plates and screws are removed if they appear to be a contributing factor. Studies have shown that early intervention with minor surgical procedures combined with pharmacological methods may improve the prognosis of ORN [51]. Surgical management is generally employed when conservative management is unsuccessful and there is progressive (Notani III) ORN resulting in pathological fractures and draining fistulae [52]. Those with more advanced ORN may require extensive surgical resections such as segmental mandibulectomy and osteo cutaneous free-flap reconstruction. Although a variety of free flaps are available for microvascular reconstructive technique, the fibula remains the workhorse for reconstruction in mandibular ORN [53]. The literature on the use of HBO for prevention or management of ORN is controversial. Based on a systematic review, there was no conclusive evidence to support the routine use of HBO for the prevention or management of ORN. However, adjunctive HBO may be considered for use on an individual basis in patients who failed response to conservative management and subsequent surgical resection [54]. There is insufficient evidence to support the use of HBO prior to dentoalveolar procedures in order to prevent ORN [55]. Based on the understanding of pathogenesis of ORN as a “radiation-induced fibro atrophic process,” a new therapeutic strategy with a combination of pentoxifylline (antifibrotic agent) and tocopherol (antioxidant) has shown promising results [56]. Although current literature supports the use of pentoxifylline in the treatment of ORN of the jaws, well-designed prospective studies are needed to further validate its true efficacy in the treatment of ORN [57].

### Risk prediction and prevention strategies

Numerous factors associated with the risk of developing ORN have been well documented in the literature [58, 59]. These can be broadly categorized into tumor-related factors, treatment- related factors and patient-related factors. Tumor-related factors include primary tumor site, size, stage, and proximity of tumor to bone. Treatment-related factors include total RT dose, RT technique, volume of irradiated mandible, dose fractionation, concurrent chemotherapy, and re-irradiation. Patient-related factors include tobacco and alcohol use, oral hygiene, dental caries, periodontal disease, and dental extractions before or after RT [37, 60]. Spontaneous ORN can also occur at radiation doses above 70Gy without any preceding dental trauma [12, 39]. Exposure of salivary glands to RT can lead to decreased salivary flow, increases risk of radiation caries leading to pulpal disease, infection and need for dental extraction both of which can trigger ORN. Gomez et al. found that maximum mandibular dose of >70Gy and a mean mandibular dose of >40Gy were associated with increased subsequent dental events and extractions after IMRT [61]. Also, mean parotid dose of >26Gy was predictive of subsequent dental caries [61].

A study from our institution showed that tumor size may be an important predictor of mandibular dose [62]. Larger (T3-T4) tumors showed mean doses ≥60Gy across the entire mandible. In contrast, RT for smaller (T1-T2) tumors showed higher prescribed doses to the molar regions (when compared to the anterior and premolar regions) and to the ipsilateral sides (when compared to the contralateral sides) [62]. With large T3-T4 base of tongue disease, the entire mandible is potentially in the field of radiation, and all mandibular teeth, irrespective of the laterality of the tumor, require evaluation regarding their long-term prognosis. It is reported that mandibular V50 and V60 values were higher for patients who developed ORN following IMRT [36]. Another study demonstrated zero to negligible radiation dose to the contralateral mandible in patients treated with PRT compared to IMRT and suggested that using PRT could presumably result in lower risk of ORN [41].

In our institution, the main strategy in mitigating ORN risk focuses on patient related factors including dental extractions before and after RT. A recent study that aimed to investigate the incidence of ORN between patients who have dental extraction before or after RT showed no statistical difference between the two groups [63]. To prevent risk for ORN, it is generally recommended to remove dental foci of infection within the RT field before RT [26]. All patients receiving head and neck RT at our institution are referred to the Dental Service for pre-treatment oral and dental evaluation. The decision to perform pre-RT dental extractions is based on several factors. Knowledge of radiation dose, treatment modality, field of radiation, and tumor prognosis play an important role in the clinical decision-making. Consideration is given to the pre-existing dental status, and extraction is indicated when there is evidence of advanced caries with poor restorative prognosis, periodontal disease, and nonfunctional teeth within the radiation field. An atraumatic approach in the extraction procedure with primary closure at the time of extraction is applied for soft tissue integrity and minimizing postoperative complications (i.e., postoperative wound healing and ORN). Adequate time for healing of extraction sites before RT is considered essential. Following the consensus report from the National Cancer Institute (NCI), we recommend a healing time of 10 to 14 days between extractions and the commencement of head and neck RT [64]. The protocol for dentate patients undergoing head and neck RT or with a history of head and neck RT also includes a prescription of neutral sodium fluoride 1.1% with 5,000 parts per million (ppm) in the form of a dentifrice toothpaste [65].

**Figure 2:**
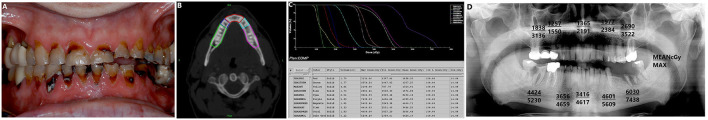
**(A)** A 55-year-old- male patient, diagnosed with HPV positive T2N2M1 squamous cell carcinoma of left palatine tonsil and treated with concurrent chemoradiation (6996cGy in 33 fractions), reported to our Dental Service for opinion and management of grossly decayed left mandibular posterior teeth. **(B)** CT slide with 5 different teeth regions contoured in the mandible. **(C)** Dose volume histogram depicting maximum and mean radiation dose to 10 different teeth region in the maxilla and mandible. **(D)** Mean and maximum dose to the different teeth regions are mapped on to the patient's panoramic radiograph. Teeth-bearing regions with prescribed dose = above 5000Gy are considered at increased risk for ORN. For example, the ipsilateral mandibular left molar region had prescribed mean and maximum doses of 6030cGy and 7438cGy, respectively. Thus, this region is believed to be at high risk for development of ORN. Our recommended treatment included endodontic therapy of tooth # 17 followed by crown amputation and maintenance of tooth #18 to allow self-exfoliation.

The major risk of ORN has been associated with post-RT dental extraction [66]. Wound healing in the mandibular posterior arches is considered compromised when dental extractions are performed in the field of radiation doses above 60Gy. Because of IMRT's complex 3-dimensional dose delivery and tissue sparing favoring the major salivary glands, different dose gradients across the mandible are created. This makes it difficult to determine the dosimetric distribution to the jaws and thus, predict areas at risk for ORN. Dosimetric contouring provides an estimate of the prescribed radiation dose to specified regions of the jaws, thus allowing the clinician to make dental treatment recommendations based on predicted risk for ORN (Figure 2). At our institution, dosimetric analysis is performed for all patients by retrieving radiation treatment planning and using calculation algorithms that incorporate tridimensional beam modeling. A dedicated dental oncologist, assisted by a medical physicist, reviews each patient's computerized treatment plans based on axial slices of computed tomography scans to calculate the cumulative dose for each group of radiated teeth. Using institutional radiation treatment planning software, the mandible in its entire height, from the alveolar crest to the inferior cortex, is manually contoured for the bone surrounding the five regions namely, right molars, left molars, right premolars, left premolars, and anterior teeth (canine to canine) for mandible and maxilla [62]. The teeth are evaluated on both the ipsilateral and contralateral sides of the primary tumor location. After selecting the five regions, the mean dose delivered to each group of teeth is determined by individually contouring teeth-bearing regions on the treatment planning systems and cumulative doses volume histograms are produced for each region. The mean and maximum point doses for each defined region are then calculated. Tsai et al. demonstrated prediction models that could also be used to estimate the maximum radiation dose to the different teeth region following RT in tonsillar cancer patients and suggested that similar methodologies can be used to generate nomograms for different disease subsites [67].

## Conclusion

Despite reduced prevalence due to advances in head and neck radiation treatment modalities, ORN remains a significant oral complication of head and neck RT. Future research directions include multi-institutional studies with large sample sizes and randomized controlled trials focused on the management of established cases. Management of ORN should focus on prevention or risk mitigation. Unfortunately, standardized preventive protocols, which may be the most effective way in reducing the risk for ORN, are lacking in the literature. In the meantime, multidisciplinary team communications, carefully planned dentoalveolar procedures pre- and post-radiation therapy and a meticulous survivorship program can reduce risk for ORN and maintain and improve quality of life in head and neck cancer patients.

## Author contributions

AS and CE: drafting of the manuscript. SY, KK, JR, and JH: revision and edits. CE: supervision. All authors have participated in the preparation of this manuscript. All authors contributed to the article and approved the submitted version.

## Funding

This study was supported, in part, by NIH/NCI Cancer Center Support Grant P30 CA008748.

## Conflict of interest

The authors declare that the research was conducted in the absence of any commercial or financial relationships that could be construed as a potential conflict of interest.

## Publisher's note

All claims expressed in this article are solely those of the authors and do not necessarily represent those of their affiliated organizations, or those of the publisher, the editors and the reviewers. Any product that may be evaluated in this article, or claim that may be made by its manufacturer, is not guaranteed or endorsed by the publisher.
